# Impact of coronavirus disease 2019 pandemic on dental student's performance across disciplines during community site rotations: A comparative analysis

**DOI:** 10.1002/jdd.13707

**Published:** 2024-09-16

**Authors:** Anubhuti Shukla, Bhavya Vaishnavi Amrutham

**Affiliations:** ^1^ Department of Dental Public Health and Dental Informatics Indiana University School of Dentistry Indianapolis Indiana USA

**Keywords:** clinical skills, community rotations, COVID‐19, dental education, interprofessional collaboration, patient management

## Abstract

**Objective:**

The coronavirus disease 2019 (COVID‐19) pandemic disrupted educational frameworks worldwide, particularly affecting health professions education. The impact on students varied based on local pandemic severity, public health policies, and institutional capacities. Our observational, retrospective study evaluated the impact of the COVID‐19 pandemic on senior dental students' performance during community rotations at Indiana University School of Dentistry.

**Methodology:**

Data from student self‐assessments and faculty evaluations, collected as part of the Community‐Based Dental Education (CBDE) course between 2018 to 2022 was utilized. Five key domains of patient care: diagnosis and treatment planning, clinical skills, time management, interprofessional collaboration, and professionalism was assesed using a 5‐point Likert scale. Pre‐ and post‐pandemic comparisons of self‐reported student evaluation and faculty assessment scores were conducted using two‐sample t‐tests, with ANOVA used to analyze cohort differences.

**Results:**

Significant declines were observed in mean scores for diagnosis and treatment planning, clinical skills, time management, and interprofessional collaboration during the post‐COVID period compared to pre‐COVID levels (p < 0.05). Conversely, an increase in average scores related to students' professionalism was noted, although this difference was not statistically significant (p > 0.05). Class cohort‐wise analysis showed similar patterns, with all cohorts experiencing significant declines in the aforementioned areas (p < 0.05) and non‐significant increases in professionalism scores (p > 0.05).

**Conclusion:**

This study findings underscore the challenges faced by dental education in adapting to pandemic‐induced disruptions and highlight the importance of adaptive approaches and support systems to address these challenges. Moving forward, resolving disparities in assessments and comprehending the various factors influencing student performance will be crucial to preserving the adaptability and effectiveness of dentistry education in the face of adversity.

## 1 INTRODUCTION

The onset of the coronavirus disease 2019 (COVID‐19) pandemic initiated an unparalleled global health emergency, significantly disrupting educational frameworks across the globe, notably within health professional educational realms such as Medicine and Dentistry. The timing of the pandemic waves and subsequent educational adjustments varied globally, leading to a diverse impact on students' experiences. Factors such as local pandemic severity, public health policies, and institutional capacities contributed to varied educational outcomes.

Prior studies have documented the pandemic's extensive impact on medical education. For instance, a study observed a decline in medical students' crucial clinical skills, alongside a decrease in self‐reported proficiency, especially in mental health skills attributable to the pandemic‐induced shift toward telehealth services and their use in medical education.^1^ Similarly, another study reported that emergency medicine rotations were notably affected by the pandemic, resulting in fewer patient interactions and varying impacts on clinical competencies.^2^ Literature also notes, the effect of pandemic‐induced disruption led to a change in medical students' career perspectives, with many expressing concerns over challenges in exploring specialties.^3^ In South Korea, the resumption of clinical rotations amidst the pandemic revealed mixed sentiments among the medical trainees regarding safety and the integrity of clinical learning, underscoring the complexities of maintaining educational quality during such crises.^4^ Pandemic‐led shutdown and social distancing measures exacerbated the difficulties in ensuring continuity and excellence of clinical training.^5^

In response to the emergence of COVID‐19, organizations such as the American Dental Association and the World Health Organization initially recommended the postponement of non‐urgent dental procedures.^6^, ^7^, ^8^, ^9^, ^10^ At the pandemic's onset, dental practices were predominantly closed or limited to providing emergency services.^11^, ^12^ A study reported an almost 50% increase in canceled appointments during the shutdown with an increasing number of patients discontinuing treatments accompanied by a decline in first‐time visits to dental clinics.^13^ This is supported by another study that notes patients avoiding routine dental treatment and opting for emergency dental treatment only, with only a limited number consenting to go ahead with procedures such as fillings, root canals, and extractions.^14^ Furthermore, COVID‐19‐induced unemployment in the United States adversely impacted individuals' access to dental care, particularly in states without expanded Medicaid or adult Medicaid dental benefits, exacerbating the lack of dental care utilization.^15^

Dental education faced significant challenges due to the intrinsic high‐risk nature of its clinical training environments. Procedures in dentistry often require close physical proximity and involve aerosol‐generating practices, elevating the risk of virus transmission.^16^ Dental school administrators faced a critical challenge in balancing the safety of students, dental educators, and patients with the uninterrupted delivery of dental education during the pandemic.^17^ With the imposed restrictions, across the United States, most dental schools halted non‐emergency clinical activities and enforced social distancing measures in preclinical simulation laboratories.^17^ Dental education's core—characterized by direct patient engagement and cultivation of practical clinical skills was, therefore, profoundly impacted.^18^ The adoption of virtual learning, especially simulation‐based training, ensured continuous learning and skill development during the pandemic.^19^, ^20^ Such adjustments necessitated not only the integration of advanced simulation models but also structured opportunities for in‐person interaction, mentorship, and feedback to cultivate essential competencies such as professionalism and communication skills.^21^ Transition to online learning significantly influenced students' interactions and led to feelings of isolation, lack of confidence with difficulty in adapting to online learning materials, which subsequently led to a negative impact on their learning abilities.^22^ The impact of quarantine on dental graduates' self‐perceived preparedness, noted students felt that they missed out on educational experiences, were less engaged and motivated, and viewed online assessments as ineffective.^23^ Therefore, despite efforts to adapt through alternative instructional methods, such as modified curricula and virtual teaching modes, these approaches proved less effective compared to traditional in‐person learning.

Continuous enhancement of educational quality necessitates ongoing assessment of its components, including inputs, outputs, processes, and outcomes. Continuous evaluation identifies gaps to allow growth and improvement.^24^ Performance assessment can take on multiple forms, including feedback from students, self‐assessment, and evaluations by department heads, colleagues, and the dean of faculty.^25^, ^26^ However, evaluations can vary due to differing perceptions. For example, studies show that student self‐assessments often rate their performance higher than teacher evaluations.^27^ This tendency for overestimation is particularly pronounced when self‐assessments contribute to the student's course grade.^27^

This study aims to evaluate the impact of the COVID‐19 pandemic on dental student's clinical competence and patient management skills during their community rotations. In addition, the study aims to compare students’ self‐reported perception of their clinical and patient management skills with supervising faculty perceptions both before and after the COVID‐induced shutdown.

## 2 METHODOLOGY

### 2.1 Study design

This observational study utilized retrospective analysis of assessment data to assess the impact of the COVID‐19 pandemic on dental students' performance across various aspects of patient care. Dental students in their senior (D4) year are required to complete a community rotation hosted at extramural clinical partner sites as part of the Community‐Based Dental Education (CBDE) Program at Indiana University School of Dentistry (IUSD). The community partner sites are federally qualified health centers and look‐alikes located in dental professional shortage areas in the state. Both self‐assessment data from senior‐year dental students and faculty assessments from community site preceptors were utilized to assess student's performance during their community rotations. This study was conducted in accordance with the guidelines and regulations set forth by the Institutional Review Board (IRB). The protocol number for this study is #: 22902, and it was determined to be exempt from full IRB review.

### 2.2 Study population

The study population included dental students enrolled in the CBDE course at IUSD between the years 2018 and 2022. The supervising dentists at community clinics were also included in the study. To measure the impact of the pandemic, data included were from 18 months before the shutdown (September 2018–March 2020) and 18 months after (September 2020–March 2022).

### 2.3 Data collection

Data for the comparative analysis of students' performance across disciplines during community site rotations was gathered from two main sources (Assessment tools):

#### 2.3.1 Faculty surveys

Faculty members, at community partner sites completed online surveys through REDcap evaluating students across various disciplines, including diagnosis and treatment planning, clinical skills, time management, and interprofessional collaboration. These surveys were administered post‐student rotation; one survey was completed for each student.

#### 2.3.2 Post‐rotation student evaluations

Senior‐year dental students participating in the clinical rotation also completed online surveys via REDcap. These surveys included self‐assessments of their performance across domains such as professionalism, as well as the above‐mentioned domains assessed in faculty surveys.

### 2.4 The five domains of patient care are described below

Diagnosis and treatment planning: The term “diagnosis,” involves four steps: collecting facts, analyzing data, correlating findings with potential diseases, and selecting the disease that best fits the patient's condition.^28^ Treatment planning involves selecting appropriate cases, evaluating the complexity of treatment for each patient, and organizing the procedures to achieve optimal health and function.^29^

Clinical skills: Includes history‐taking, physical exams, clinical investigations, diagnostic reasoning, procedural accuracy, effective communication, teamwork, and professionalism.^30^ In our surveys, the clinical skill assessment included: oral surgery; operative, radiology, pediatric dentistry, periodontics, endodontics, caries risk assessment, administration of local anesthetic, and pain management. The scores used for comparison in the study represent a cumulative average of all these specified disciplines

### 2.5 Time management: Time management is defined as the process of making decisions to organize, safeguard, and adjust one's time in response to changing circumstances.^31^

Interprofessional collaboration: Interprofessional collaboration is a partnership between health providers and clients that involves shared decision‐making on health and social issues.^32^ This collaborative practice, characterized by communication, responsibility, accountability, coordination, cooperation, assertiveness, autonomy, and mutual trust and respect, allows a synergistic use of collective knowledge and skills.^33^

Professionalism: Professionalism encompasses the consistent and skillful use of communication, knowledge, technical abilities, clinical reasoning, emotions, values, and reflection in daily practice, aiming to benefit both individuals and communities served.^34^

A 5‐point Likert scale was employed to assess students' skills in various disciplines. The scale is structured as follows:

1. Unsatisfactory
2. Needs improvement
3. Meets expectations
4. Satisfactory
5. Exceeds expectations

Both faculty and students used these numerical ratings to indicate the proficiency levels across the evaluated skills.

The assessment tools for students and supervising dentists are included in Appendix I.

### 2.6 Data analysis

Descriptive statistics for student demographics were computed. Two‐sample assuming unequal variances was conducted using Excel's Data Analysis Toolpak to compare student self‐reported scores and faculty assessment scores before and after the COVID‐19 period. In addition, a class cohort‐wise comparison was performed using analysis of variance (ANOVA) to assess differences across various class years within the same time frame (2019–2022).

## 3 RESULTS

The study included a total of 40 community site faculty, supervising the student performance during their community clinical rotation, and 350 student respondents, which included a total of 181 females (52%) and 168 males (48%). Among the student respondents, the majority identified as White or Caucasian (69.3%). In terms of age distribution, 77% were aged 30–39 years, as illustrated in Table 1.

**TABLE 1:** Student demographics.

Sex	Race/Ethnicity	Age groups
Female (n = 181, 52%)	White or Caucasian (*n* = 242, 69.3%)	20–29 Years (*n* = 53, 16%)
Male (*n* = 168, 48%)	Asian (*n* = 77, 22%)	30–39 Years (*n* = 254, 77%)
	Hispanic or Latino (*n* = 8, 2.2%)	40–49 Years (*n* = 22, 6.6%)
	Black or African American (*n* = 9, 2.5%)	50–59 Years (*n* = 1, 0.3%)
	Multicultural (*n* = 13, 3.7%)	

The results revealed significant differences between student and faculty scores, with higher mean scores observed in the pre‐COVID period across all categories, as determined by a two‐sample T‐test. ANOVA analysis for year‐wise comparisons from 2019 to 2022 showed statistical differences in scores across different academic years within each discipline. The detailed comparisons of pre‐ and post‐COVID scores, as well as the year‐wise analysis and statistical significance for each category, are presented in Tables 2 and 3, respectively.

**TABLE 2:** Faculty and student assessment scores: T‐test pre‐ and post‐COVID.

Skills	Group	Pre‐COVID mean	Pre‐COVID variance	Post‐COVID mean	Post COVID Variance	P(T ≤ t) two‐tail	t Critical two‐tail
**Diagnosis and Treatment Planning**	**Student**	4.45	0.0469	4.02	0.6693	1.67E‐13	1.969
	**Faculty**	4.46	0.0298	4.14	0.5518	9.47E‐13	1.969
**Clinical skills**	**Student**	4.4	0.0426	3.94	0.4419	3.22E‐25	1.9684
	**Faculty**	4.4	0.0459	4.16	0.4064	5.98E‐13	1.9662
**Time management**	**Student**	4.43	0.0663	4.10	0.8393	2.79E‐07	1.9688
	**Faculty**	4.44	0.0574	4.07	0.6695	1.06E‐12	1.9665
**Interprofessional collaboration**	**Student**	4.49	0.0088	4.20	0.7965	1.92E‐06	1.9703
	**Faculty**	4.46	0.0296	4.35	0.6366	0.0117	1.9671
**Professionalism**	**Student**	4.49	0.0065	4.52	0.5118	0.440783	1.9696

**TABLE 3:** Class cohort year‐wise comparison of student and faculty mean scores across disciplines (2019–2022) with analysis of variance (ANOVA) results.

Discipline	Measure	2019	2020	2021	2022	*p*‐value (Student)	*p*‐value (Faculty)
**Diagnosis and treatment planning**	**Student mean score**	4.40	4.46	4.00	3.97	4.18E‐08	2.31E‐07
	**Faculty mean score**	4.47	4.46	4.23	4.11		
**Clinical skills**	**Student mean score**	4.43	4.47	3.88	3.97	1.94E‐17	3.83E‐13
	**Faculty mean score**	4.47	4.44	4.23	3.95		
**Time management**	**Student mean score**	4.43	4.43	4.01	4.31	2.23E‐05	1.51E‐08
	**Faculty mean score**	4.45	4.42	4.15	3.93		
**Interprofessional collaboration**	**Student mean score**	4.50	4.47	4.20	4.15	0.0017	0.33
	**Faculty mean score**	4.47	4.48	4.39	4.35		
**Professionalism**	**Student mean score**	4.50	4.48	4.44	4.66	0.0578	

### 3.1 Diagnosis and treatment planning

Faculty mean scores for Diagnosis and Treatment Planning were 4.47 in the pre‐COVID phase and decreased to 4.14 in the post‐COVID phase. This decrease was found to be statistically significant (*p* < 0.05). Similarly, student mean scores also decreased from 4.45 in 2018‐2020 to 4.02 in 2020‐2022, also showing a significant decline (*p* < 0.05). Faculty scores slightly surpassed students' self‐reported scores both before and after the COVID‐19 pandemic. ANOVA results showed a general decline in mean scores over the years for different class cohorts (2019–2022), with the Class of 2019 averaging 4.40, to a score of 3.97 for the Class of 2022. Faculty scores also decreased from 4.47 for the Class of 2019 to 4.11 for the Class of 2022 (*p* < 0.05).

### 3.2 Clinical skills

Similarly, faculty mean scores for Clinical Skills were 4.45 from 2018 to 2020 and decreased to 4.16 from 2020 to 2022 indicating a statistically significant decline (p < 0.05). Similarly, student mean scores dropped significantly from 4.46 to 3.94 (*p* < 0.05), as shown in Table 2. During the pre‐COVID period, both faculty and students had identical mean scores. Post‐COVID, although both faculty and student scores decreased, faculty scores remained higher than student scores in this category as well. Additionally, cohort‐wise results indicated that both student and faculty scores decreased over time, starting at 4.43 for the Class of 2019 to 3.97 for the Class of 2022. Faculty scores similarly decreased from 4.47 in 2019 to 3.95 in 2022. These changes in scores are statistically significant, with *p*‐values < 0.05.

### 3.3 Time management

Faculty mean scores for Time Management decreased significantly from 4.44 in 2018–2020 to 4.07 in 2020–2022 (*p* < 0.05). Similarly, student mean scores decreased from 4.43 in 2018–2020 to 4.10 in 2020–2022, also showing a statistically significant decline (*p* < 0.05). Pre‐COVID, faculty mean scores were marginally higher than students' self‐reported scores, whereas post‐COVID, faculty‐assigned scores were slightly lower than students' self‐reported scores. Cohort‐wise results revealed that student mean scores declined from 4.43 for the class of 2019, to 4.01 for the class of 2021, and then increased to 4.31 for the class of 2022. In contrast, faculty scores declined from 4.45 in 2019 to 3.93 in 2022. Both student and faculty scores show significant variations over time, with *p*‐values < 0.05.

### 3.4 Interprofessional collaboration

Faculty mean scores for Interprofessional Collaboration decreased from 4.46 in 2018‐2020 to 4.35 in 2020–2022, with a statistically significant difference (*p* < 0.05). Similarly, student mean scores decreased from 4.49 in 2018–2020 to 4.2 in 2020–2022, indicating a significant decline (*p* < 0.05). Pre‐COVID, student self‐reported scores were slightly higher than faculty‐assigned scores; however, post‐COVID, faculty scores remained higher compared to students' self‐reported scores. Cohort‐wise results showed a slight decrease in student scores across different class cohorts, dropping from 4.50 for the class of 2019 to 4.15 for the class of 2022. In comparison, faculty scores declined from 4.47 in 2019 to 4.35 in 2022. The changes in student scores are significant (*p*‐value < 0.05), while faculty scores show less significant differences (*p*‐value > 0.05).

### 3.5 Professionalism

Comparing students' professionalism scores before and after COVID‐19, the average score increased slightly from 4.49 to 4.52 post‐pandemic. Similarly, class‐wise ANOVA results showed that student mean scores were 4.50 for the class of 2019, increasing to 4.66 for the class of 2022. However, in both cases, the differences were not statistically significant, with *p*‐values > 0.05.

## 4 DISCUSSION

Our study demonstrates a significant decline in both faculty‐assigned as well as student self‐reported scores across key disciplines of patient care such as diagnosis and treatment planning, clinical skills, time management, and interprofessional collaboration, post‐COVID‐19 shutdown. Our study findings can be attributed to the negative impact of the pandemic which largely restricted pre‐clinical and clinical training due to several reasons: insufficient infrastructure, insufficient patient experiences, and social distancing measures. All of these and more resulted in reduced opportunities for clinical practice, patient management, time management, and collaborative learning experiences essential for comprehensive education, which later translated to their performance at extramural rotations in the senior year.

In the Diagnosis and Treatment Planning domain, faculty consistently scored slightly higher than students both before and after the COVID‐19 pandemic, with declines observed in scores for both groups post‐COVID. Analyzing scores across different graduating classes, from the class of 2019 to the class of 2022, reveals a decrease in scores. Specifically, the class of 2022, which was in its preclinical phase during the pandemic, likely experienced disruptions in foundational/preclinical training, potentially affecting their clinical performance in their final year and resulting in lower scores.

In terms of clinical skills, faculty and students had identical mean scores before COVID‐19, and both groups experienced a decline in scores post‐COVID. The trend observed in cohort‐wise analysis confirmed a decline in scores between the Class of 2019 and 2022 with significant statistical differences. The decrease in clinical skills scores may be attributed to the differential impact of the pandemic on various cohorts. The insufficient preparation during their early years of dental education could have compounded over time, leading to observable deficiencies in their final year when they were expected to perform more advanced clinical tasks. Similar to our study, Radović et al. studied the self‐confidence levels of 2020 final‐year dental students, finding that those who participated in distance learning due to the COVID‐19 shutdown had significantly lower self‐confidence in performing clinical procedures compared to the 2019 graduates, who benefited from traditional in‐person education and direct patient interaction.^35^ The significant decline in self‐confidence attributed to a lack of hands‐on experiences, communication issues, and ever‐changing policies, was particularly evident in disciplines taught during the later semesters, which coincided with the pandemic shutdown.^35^ Students who were in the initial years of the training program, also had their pre‐existing skills impacted by the lack of further refinement opportunities, with variations in clinical opportunities for hands‐on practice.^35^ Another study examining the supervision of radiography students by clinical tutors during the COVID‐19 pandemic, revealed that a substantial proportion of respondents perceived COVID‐19 as negatively impacting the students' clinical practical experience.^36^

Our study results noted a decline in time management scores from 2019 to 2021, suggesting the effect of the pandemic. The class of 2022, who experienced online learning during their preclinical years, showed a partial recovery in their scores; however, these scores remained lower compared to earlier cohorts. The transition to online learning along with the lack of direct communication and support, made it challenging for students to regulate their time and maintain focus on their academic responsibilities, despite their efforts to adapt and reestablish academic routines.^37^ ^–^ ^38^ Additionally, the cancellation of clinical assessments and patient interactions significantly hampered their practical training and time management skills​.^39^ Consistent with our study findings, a study by Aguilera‐Hermida, noted a decline in self‐reported time management skills of students during the pandemic.^37^ Another study examining the shift from traditional to online learning during the COVID‐19 pandemic found that nearly half (48%) of the participants expressed concerns about their time management skills.^38^

In the area of Interprofessional Collaboration, pre‐COVID, student self‐reported scores were slightly higher than faculty‐assigned scores. However, post‐COVID, while both faculty and student scores comparatively decreased, faculty scores consistently remained higher than students.

Supporting these findings, our class cohort‐wise analysis revealed a decline in student scores for Interprofessional Collaboration from 2019 to 2022. Similar to our study, a case report detailing the interprofessional collaborative practice encounters of three acute care physical therapists during the COVID‐19 pandemic highlighted challenges and opportunities that corresponded with the four interprofessional educational competencies, for example, values/ethics, responsibilities, communication, and teamwork.^40^ Various challenges like role confusion and communication issues collectively diminished effective interprofessional collaboration during the pandemic.^40^ Additionally, evolving and inconsistent pandemic policies, coupled with the sudden adoption of telehealth and the formation of new, less cohesive teams, led to fragmented care.^40^ In contrast to our study findings, a comparative study involving pharmacy and medical students revealed that those participating in virtual interprofessional education (IPE) learning activities during the pandemic reported greater perceived gains in interprofessional skills, as well as development in professionalism, interpersonal communication skills, and practice‐based learning, compared to those in pre‐COVID‐19 in‐person clinical home visits.^41^ The reasons cited for the effectiveness of the virtual IPE activity include its concentrated focus on IPE objectives and the enhanced collaboration opportunities it provided, avoiding the distractions of complex in‐person factors such as patients' socioeconomic contexts.^41^ The above presents educators with an opportunity that can be utilized to make curricular changes and implement interprofessional collaborative experiences.

Class‐wise analysis of scores in professionalism between 2019 to 2022, showed classes of 2020 and 2021 had reduced scores, while the class of 2022 achieved the highest professionalism scores. This can be explained by the fact that the class of 2022, being in their preclinical years during the pandemic, faced fewer disruptions in their foundational coursework compared to those in direct patient care. In contrast, the classes of 2020 and 2021, who were in their final years during the pandemic, experienced significant limitations in daily patient interactions. This restricted their ability to develop and demonstrate professionalism through communication, clinical reasoning, and skill application. A study by Prade et al noted medical students' views on professionalism remained largely unchanged during the pandemic.^42^ However, participants did acknowledge an enhanced emphasis on qualities such as maintaining composure, improving communication skills, and enhancing professional competencies.^42^ These findings collectively indicate a nuanced adaptation that highlights the context‐sensitive nature of professionalism, showing how external factors like the pandemic shape perceptions and priorities, and illustrates how the emotional strain on students and changes in their motivation and career aspirations deeply impact professional identity.^42^

Our study results emphasize the significance of efficient feedback systems in connecting self‐evaluation with standardized assessment. Variations between evaluations by faculty members and self‐assessments by students in all disciplines hint at a possible discrepancy in how students perceive their skills in these key areas, either due to a lack of confidence in their clinical abilities and diagnostic skills or inexperience in self‐assessment, resulting in lower self‐assessment.^43^, ^44^ In line with our study results, a study conducted to evaluate the impact of new COVID‐19 pandemic protocols on student performance and self‐evaluation abilities found that students during the COVID‐19 pandemic received higher average faculty scores across all four preclinical laboratory competency assessments and in the final multiple‐choice examination.^45^ Furthermore, the nature of the discipline itself may influence how faculty and students perceive performance.^46^, ^47^ Faculty members may prioritize certain aspects of these skills that students may not fully recognize or appreciate, leading to differences in evaluations.^46^, ^47^ Additionally, the impact of the COVID‐19 pandemic on students' mental health and well‐being, including stress and uncertainty, could have influenced their ability to feel as confident in their skills, potentially contributing to differences observed in their self‐reported scores in these areas.^48^

In contrast to our findings, a meta‐analysis on medical students' self‐assessment accuracy found that students moderately tend to overestimate their abilities in communication‐focused, standardized patient interactions.^49^ Another study comparing assessment scores of second‐ and third‐year dental students with faculty evaluations in preclinical restorative dentistry found that second‐year students tended to overestimate their skills and considered themselves to be above average when compared to their third‐year peers.^50^ This difference in assessment presents an interesting area to further explore and find opportunities that could lead to improved student outcomes.

Our study presents important findings about aspects of patient care that were significantly affected due to the pandemic and the resultant student performance. Limitations of the study arise from self‐reported assessment scores from students rather than objective measures of competence, suggesting a subjective viewpoint. Even the faculty‐assigned scores may have a component of subjectivity in spite of appropriate calibration. Additionally, the study's cross‐sectional design, spanning over a limited time frame and limited to data from one dental school, limits the generalizability of the study results.

## 5 CONCLUSION

Our study reveals a notable decrease in both faculty‐assigned and student‐self‐reported scores across patient care disciplines following the COVID‐19 pandemic, emphasizing its impact on dental education. The pandemic impacted students at different levels of training, depending on what class cohort they belonged to, and this resulted in differences in different aspects of patient care. The study highlights shared challenges among students and faculty, emphasizing the need for adaptive approaches and support systems to address disruptions to traditional learning environments. Moving forward, addressing evaluation discrepancies and acknowledging diverse influences on student performance will be crucial for maintaining the resilience and efficacy of dental education in the face of ongoing challenges.

## Supporting information

Supporting Information
