# Supplementary material for: Impact of coronavirus disease 2019 pandemic on dental student's performance across disciplines during community site rotations: A comparative analysis
Source: J Dent Educ. 2024 Sep 16;89(2):145–54. doi: 10.1002/jdd.13707 (PMC11862985; doi:10.1002/jdd.13707)
Supplement: Supplementary file 1 — Supporting Information [file JDD-89-145-s001.docx]

**Appendix 1**

1. **Post-Rotation Student Evaluations:**

**Impact of CBDE on preparedness for clinical practice**

**Preparedness Prior to CBDE: Reflecting back to before you completed the CBDE Rotation, how prepared were you for the CBDE rotation?**

1: Unsatisfactory

2: Needs Improvement

3: Meets Expectations

4: Satisfactory

5: Exceeds Expectations

**Please indicate whether and to what extent the CBDE rotation experience has prepared you in the following areas using the above scale (1 to 5):**

1. **Diagnosis and Treatment Planning: Ability to independently diagnose and treatment plan utilizing critical thinking and problem solving skills, including emergency treatment situations.**
2. **Clinical Skills: Ability to approach clinical care with confidence, efficiency, and independence; ability to recognize procedures that are within and beyond scope of care; ability to adapt and respond appropriately to evolving clinical situations; ability to adhere to infection control standards. Please rate your performance or preparedness in the following clinical skill categories based on the scale provided (1 to 5);**
3. Oral Surgery
4. Operative
5. Radiology
6. Pediatric Dentistry
7. Periodontal Procedures
8. Caries Risk Assessment
9. Endodontics
10. Administration of Local Anesthetic
11. Pain Management
12. **Time management: Ability to manage patient care within scheduled time allotment; ability to be flexible with scheduling to accommodate emergency appointments and/or walk in patients.**
13. **Interprofessional Collaboration: Ability to communicate effectively with other healthcare providers, demonstrate effective teamwork skills, integrate the abilities of team members to optimize health and provision of care and reflect on self and team practices to develop strategies that enhance teamwork.**
14. **Professionalism: Ability to communicate respectfully with other medical professionals and community members outside the healthcare team; ability to be punctual; ability to maintain a professional appearance and adhere to clinical dress code (including wearing name badge); ability to maintain a professional demeanor during times of unexpected outcomes.**
15. **Faculty Survey:**

**How prepared did you feel the student was in the following areas during their community site rotations? Please rate the student’s ability on a scale of 1 to 5.**

1: Unsatisfactory

2: Needs Improvement

3: Meets Expectations

4: Satisfactory

5: Exceeds Expectations

1. **Diagnosis and Treatment Planning: Ability to independently diagnose and treatment plan utilizing critical thinking and problem solving skills, including emergency treatment situation**
2. **Clinical Skills: Ability to approach clinical care with confidence, efficiency, and independence; ability to recognize procedures that are within and beyond scope of care; ability to adapt and respond appropriately to evolving clinical situations; ability to adhere to infection control standards.**
3. Oral Surgery
4. Operative
5. Radiology
6. Pediatric Dentistry
7. Periodontal Procedures
8. Caries Risk Assessment
9. Endodontics
10. Administration of Local Anesthetic
11. Pain Management
12. **Time management: Ability to manage patient care within scheduled time allotment; ability to be flexible with scheduling to accommodate emergency appointments and/or walk in patients.**
13. **Interprofessional Collaboration: Ability to communicate effectively with other healthcare providers, demonstrate effective teamwork skills, integrate the abilities of team members to optimize health and provision of care and reflect on self and team practices to develop strategies that enhance teamwork.**
